# Influence of systemic fluoroquinolone administration on the presence of *Pasteurella multocida *in the upper respiratory tract of clinically healthy calves

**DOI:** 10.1186/1751-0147-50-36

**Published:** 2008-09-22

**Authors:** Boudewijn Catry, Siska Croubels, Stefan Schwarz, Piet Deprez, Bianca Cox, Corinna Kehrenberg, Geert Opsomer, Annemie Decostere, Freddy Haesebrouck

**Affiliations:** 1 Department of Reproduction, Obstetrics and Herd Health, Faculty of Veterinary Medicine, Salisburylaan, 133, Ghent University, 9820 Merelbeke, Belgium; 2 Department of Internal Medicine and Clinical Biology of Large Animals, Faculty of Veterinary Medicine, Salisburylaan, 133, Ghent University, 9820 Merelbeke, Belgium; 3 Department of *Pharmacology, Toxicology and Biochemistry*, Faculty of Veterinary Medicine, Salisburylaan, 133, Ghent University, 9820 Merelbeke, Belgium; 4 Department of Pathology, Bacteriology, and Poultry Diseases, Faculty of Veterinary Medicine, Salisburylaan, 133, Ghent University, 9820 Merelbeke, Belgium; 5 Scientific Institute of Public Health, Rue Juliette Wytsmanstraat 14, 1050 Brussels, Belgium; 6 Institute of Farm Animal Genetics, Friedrich-Loeffler-Institute (FLI), Hoeltystr. 10, 31535 Neustadt-Mariensee, Germany

## Abstract

The influence of enrofloxacin administration (5 mg/kg) for five consecutive days on the occurrence of *Pasteurella multocida *in the upper respiratory tract of two healthy calves was monitored over a 10-day period. From nasal swabs of two additional healthy control calves, which received a placebo saline administration, *P. multocida *was isolated throughout the study period. In the enrofloxacin treated calves, *P. multocida *was not demonstrated in the nasopharynx from 48 h after the first injection until two days after the last administration, when *P. multocida *reappeared and proved to be clonal in nature to the original isolates. During the experiment, no change in minimal inhibitory concentration for enrofloxacin of the *P. multocida *isolates was detected (MIC ≤ 0.015 μg/mL). Enrofloxacin concentrations were determined in the plasma by a high-performance liquid chromatography method with fluorescence detection. The PK/PD indices AUC/MIC and C_max_/MIC ratio were calculated and found to be 1157.7 and 129.8, respectively. Remarkably, the respiratory pathogen *Arcanobacterium pyogenes *became the predominant recovered organism in the nasopharynx of one animal following enrofloxacin therapy throughout the remaining of the experiment.

## Findings

In calves, enrofloxacin is frequently used to treat pneumonic pasteurellosis, a disease mostly due to *Pasteurella multocida *[1]. *P. multocida *is a common inhabitant of the upper respiratory tract of calves. To better understand the epidemiology of pneumonic pasteurellosis and the occurrence of antimicrobial resistance, knowledge is needed on how systemic fluoroquinolone administration affects the flora of the nasopharynx in healthy calves. This is important as metaphylaxis is a common practice in the prevention of bovine respiratory diseases. Preventive treatment of "at risk animals" may be associated with a selection pressure leading to antimicrobial resistance or a shift in the population of bacteria present in the nasopharynx.

The aim of the present experiment was to evaluate the influence of consecutive systemic enrofloxacin administrations on the presence and susceptibilities of *P. multocida *strains naturally present in the nasopharynx of clinically healthy calves and to find a relationship with pharmacokinetic/pharmacodynamic surrogate parameters.

Four dairy calves aged 24–28 days were loose group-housed together during a 5-day pre-experimental period after which the calves were randomly assigned to either a treatment group or a control group. Inclusion criteria were the absence of disease and antimicrobial therapy since birth. The calves were housed two by two in straw-bedded pens (approximately 18 m^2 ^of floor space per group) in the same automated ventilated stable (17 ± 2°C), but divided by full wooden partitions approximately 1.4 m high. Management and hygienic measures were set up to prevent direct contact between the two study groups. Water and hay were supplied *ad libitum*, and the calves were maintained on an antibiotic-free milk replacer diet twice a day. Clinical observations were carried out and all calves remained healthy during the entire experiment.

The study lasted for 10 days (D0–D9). For five consecutive days (D0–D4), the two calves of the treatment group (weights at D0: 47.0 and 49.2 kg) were injected intramuscularly with 5 mg/kg enrofloxacin (Baytril 2.5%, Bayer, Milan, Italy), while the two calves of the control group (weights at D0: 48.7 and 50.0 kg) received a placebo (5 mL isotonic saline intramuscularly). After disinfection of the nostrils with 90% ethanol, nasal samples were collected using cotton swabs inserted 10–15 cm into the dorsal conchae (Venturi Transsystem, Copan, Italy) every 12 h starting from D0 until D5 and on D6 and D9. Samples were cooled (< 7°C) and further processed within 24 h, starting by vortexing each swab in 3 mL phosphate buffered saline for 10 s. Plasma samples obtained by centrifugation of blood at 4 × g for 10 min were taken on D0 at 0, 2, 4, 6, 12, 24 h, on D1 at 48 h, on D2 at 72 h, on D3 at 96 h, and on D4 at 100, 104, 108, 112, 120, 144 and 216 h (the latter intensity to explore the elimination phase) from the treated calves and from the placebo calves at 0 h (D0), and stored at -20°C prior to assay. The experimental protocol was approved by the local ethics committee.

**Figure 1:**
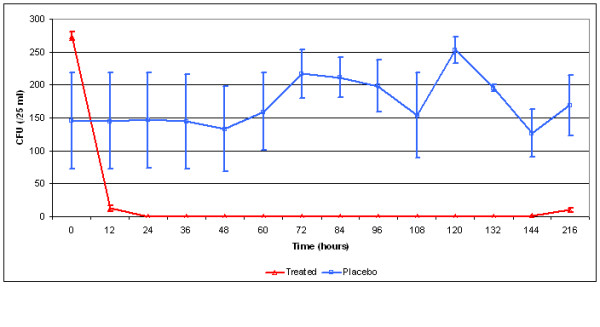
**Recovery of *Pasteurella multocida *(colony forming units, CFU) in the nasopharynx of calves treated with enrofloxacin and control calves on media without enrofloxacin.** Error bars indicate median and interquartile range.

In the nasal samples, the numbers of enrofloxacin resistant *P. multocida *isolates and the total numbers of *P. multocida *isolates were determined using a comparative enumerating procedure (duplicate aliquots of 25 μL) on Columbia agar (Oxoid, Hampshire, UK) to which sheep blood (5% vol/vol) and 16 μg/mL bacitracin (1 μg equals 0.0654 U, Sigma Poole, UK) was added with the following concentrations of enrofloxacin (Baytril 2.5%): 0; 0.06; 0.125; 0.25; 0.5, and 1 μg/mL. Reading was performed after 24 h and 48 h of aerobic incubation at 37°C, and consisted of counting colony-forming units (CFU) distributed over each drop zone and averaged for duplicates. The species identification of one *P. multocida *colony per animal per day was confirmed by means of phenotyping and tDNA-PCR and clonality was examined by means of pulsed-field gel electrophoresis (PFGE) [1]. Bacteriological counts were expressed as median and interquartile range, and a non-parametric multivariate analysis of variance (nonparametric MANOVA) for repeated measurements and small sample sizes [2] was performed (SAS version 9.1, Sat Institute Inc., Cary, NC). Evolution of *P. multocida *recovery from the nasal swabs on media without enrofloxacin in both the treated calves and the control calves is given in Figure 1. The non-parametric MANOVA showed that the difference in *P. multocida *isolation was significant between the treatment and the placebo group over time (treatment*time *P *= 0.04, time *P *= 0.03). *P. multocida *was not recovered during the entire experiment on media containing any of the enrofloxacin concentrations. Additional susceptibility testing [1] (range 0.015–1 μg/mL) confirmed no detectable increase in enrofloxacin MIC (≤ 0.015 μg/mL) of seven *P. multocida *isolates recovered from both treated calves on D0 (2) and on D9 (2) and from the control calves on D0 (1) and D9 (2), and PFGE fingerprinting patterns were identical. Whether the clonally identical organisms reappeared in the treated calves either through airborne transmission from the control calves or endogenously via undetected strains, e.g. in the tonsils, is unclear.

To evaluate whether the microbiological effects of fluoroquinolone administration were in line with the current understanding of pharmacokinetic/pharmacodynamic (PK/PD) relationships, two PK/PD parameters were calculated: the maximum plasma concentration/minimal inhibitory concentration (C_max_/MIC) ratio and the area under the inhibitory curve (AUC/MIC). Theoretically, C_max_/MIC should exceed 10 and AUC/MIC (AUIC) should exceed 125 to minimize the selection for resistant organisms by bacterial killing also of less susceptible subpopulations (eradication) [3,4].

Plasma concentrations of enrofloxacin and its active metabolite ciprofloxacin were determined using a validated high-performance liquid chromatography method (HPLC) with fluorescence detection. Extraction was performed as described by Manceau *et al. *[5], with minor modifications. Pharmacokinetic analysis was performed using MW/Pharm software (version 3.60, Medi Ware, Utrecht, The Netherlands). The plasma concentration-time profile could be adequately fitted to a one compartmental model (r^2 ^≥ 0.996 for enrofloxacin and r^2 ^≥ 0.973 for ciprofloxacin). All concentrations in the placebo calves were below the limit of detection (4.6 ng/mL). Maximal plasma concentration (C_max_), elimination rate constant (k_e_) and elimination half-life (T_1/2e_) were derived from the model. The area under the curve from time zero to infinity after the first dose (AUC_0→∞_) was calculated using the linear trapezoidal method for AUC_0→t _and adding the estimated terminal portion of the curve (Ct/k_e_), where t is the last time of measurable plasma concentrations after the first dose. Enrofloxacin is de-ethylated into ciprofloxacin, but the degree of this metabolic process substantially varies within animal species. The mean ratio in AUC_0→∞ _of ciprofloxacin/enrofloxacin after the first dose found here was 12.3%. This was significantly lower than reported in 8-month-old buffalo calves (27%) and adult cattle (29.9%) [6]. In newborn and one-week-old calves the ciprofloxacin/enrofloxacin ratio can range from 10 to 27%. The ratio is probably lower in young calves due to the lower metabolic capacity at this age [7]. The area under the concentration-time curve at steady-state over 24 h (AUC_0→24 h_) was set equivalent to the AUC_0→∞ _after the first dose. The ratio of AUC_0→24 h_/MIC (AUIC) and plasma C_max_/MIC was expressed as a dimensionless value. For the isolated *P. multocida *strains the mean AUIC and C_max_/MIC for enrofloxacin were found to be 1157.7 and 129.8, respectively (MIC for enrofloxacin ≤ 0.015 μg/mL). Even when a conservative MIC of 0.06 μg/mL [1] is taken into account, the thresholds would successfully be exceeded (289.4 for AUIC; 32.4 for C_max_/MIC). Unfortunately, the obtained values rely on the plasma concentrations and not on concentrations measured in the nasopharynx. Nevertheless, several studies dealing with pharmacokinetics of fluoroquinolones in both plasma and at the site of infection are available in cattle and in line with our observations [8,9]. Recently, it has been shown that fluoroquinolones are a substrate for ATP-dependent efflux transporters which may result in effective drug concentrations in luminal compartments of target tissues [10,11] In addition, during natural courses of bovine respiratory disease, the PK/PD surrogate markers for fluoroquinolones can largely exceed those seen in apparently healthy animals [12].

In one calf of the enrofloxacin treated group, a quasi pure culture of *Arcanobacterium pyogenes *was recovered from D2 (2.9 log10 CFU/mL) onwards and increased in numbers (up to 4.5 log10 CFU/mL at D4) to remain persistent during the remaining time of the experiment. *A. pyogenes *was identified as previously described[13] and the occurrence was observed on the selective media containing ≤ 0.25 μg/mL enrofloxacin. The latter is in agreement with the study of Yoshimura *et al. *[14] who found a MIC of 0.5 μg/mL for *A. pyogenes *and in accordance with a report by Narayanan *et al. *[15], that support our finding that bovine *A. pyogenes *strains are able to grow on the selective media containing 16 μg/mL bacitracin (equals approximately 1 U/mL). *A. pyogenes *is an opportunistic bovine pathogen associated with chronic manifestations of bovine respiratory disease.

In conclusion, a temporary eradication effect of enrofloxacin for *P. multocida *in the nasopharynx of treated calves was present. This is in line with the current PK/PD approach to prevent the selection of resistance, since the AUIC and the C_max_/MIC ratio measured in the present study largely exceeded the generally accepted thresholds of 125 and 10, respectively. Although confirmation is needed, our results suggest that other respiratory pathogens like *A. pyogenes*, which are intrinsically less susceptible for enrofloxacin, are able to colonise the upper respiratory tract during fluoroquinolone therapy.

## Competing interests

The authors declare that they have no competing interests.

## Authors' contributions

BC conceived the study and drafted the manuscript. PD, GO, AD, and FH participated in the design and coordination of the study. BC, AD and FH performed the microbiological analysis, except for the PFGE and confirmatory identification test, which were done by CK and SS. SC carried out the HPLC, pharmacokinetic/pharmacodynamic analysis, and substantially helped to draft these sections in the manuscript. BC performed and interpreted the statistical analysis. All authors read and approved the manuscript.
